# Supplementary material for: The interactome of intact mitochondria by cross-linking mass spectrometry provides evidence for coexisting respiratory supercomplexes
Source: Mol Cell Proteomics. 2017 Dec 8;17(2):216–32. doi: 10.1074/mcp.RA117.000470 (PMC5795388; doi:10.1074/mcp.RA117.000470)
Supplement: Supplemental Data [file supp_RA117.000470_133922_0_supp_23978_fzffwf.zip › spectra_annotation/mito_DR_spectra_annotation/43-1-7-1-1-1.pdf]

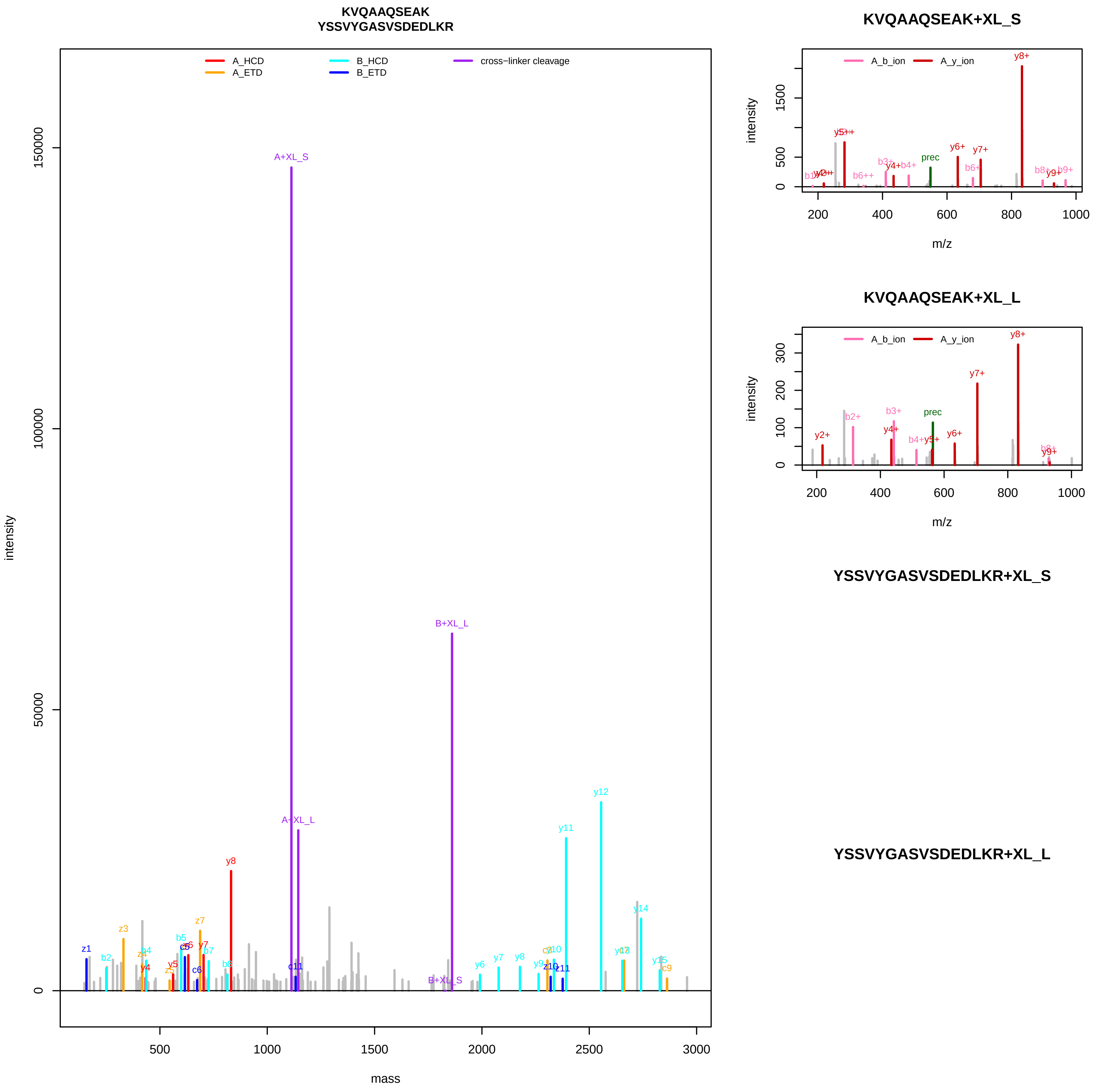
KVQAAQSEAK
YSSVYGASVSDEDLKR
A_HCD
A_ETD
B_HCD
B_ETD
cross−linker cleavage
A+XL_S
A+XL_L
B+XL_L
B+XL_S
intensity
mass
0
50000
100000
150000
500
1000
1500
2000
2500
3000
y8
y12
y11
y14
z1
b2
z3
z7
b5
c5
y6
y7
b7
b8
c6
y5
y4
z4
b4
z5
c11
y6
y7
y8
y9
c9
z10
z11
y10
y13
y15
KVQAAQSEAK+XL_S
A_b_ion
A_y_ion
y8+
y5++
y6+
y7+
y4+
b3+
b4+
prec
b6++
b6+
b8+
y9+
b9+
intensity
m/z
0
500
1500
200
400
600
800
1000
KVQAAQSEAK+XL_L
A_b_ion
A_y_ion
y8+
y7+
b3+
prec
b2+
y2+
y4+
b4+
y5+
y6+
y9+
intensity
m/z
0
100
200
300
200
400
600
800
1000
YSSVYGASVSDEDLKR+XL_S
YSSVYGASVSDEDLKR+XL_L
